# Editorial: Consciousness in Humanoid Robots

**DOI:** 10.3389/frobt.2019.00017

**Published:** 2019-03-22

**Authors:** Antonio Chella, Angelo Cangelosi, Giorgio Metta, Selmer Bringsjord

**Affiliations:** ^1^RoboticsLab, Department of Industrial and Digital Innovation, University of Palermo, Palermo, Italy; ^2^Cognitive Robotics and Social Sensing Laboratory, ICAR-CNR, Palermo, Italy; ^3^School of Computer Science, The University of Manchester, Manchester, United Kingdom; ^4^iCub Facility, Istituto Italiano di Tecnologia, Genova, Italy; ^5^Department of Computer Science, Rensselaer Polytechnic Institute, Troy, NY, United States; ^6^Department of Cognitive Science, Rensselaer Polytechnic Institute, Troy, NY, United States

**Keywords:** robot consciousness, robot awareness, machine consciousness, artificial consciousness, autonomic robotics

Building a conscious robot is a grand scientific and technological challenge. Debates about the possibility of conscious robots and the related positive outcomes and hazards for human beings are today no longer confined to philosophical circles.

There is no accepted definition of consciousness: see Vimal (2009) for an overview of different meanings of the word. However, it is useful to point out the distinction of consciousness as experience and consciousness as function. From the point of view of experience, a subject is conscious when she feels visual experiences, bodily sensations, mental images, emotions (Chalmers, 1995). As Nagel (1974) points out, a subject has conscious experience if there is something it is like to be that subject. From the point of view of function, a conscious subject is able to process information which is globally available (Dehaene et al., 2017), she integrates information (Tononi, 2008), she is introspectively aware of herself (Floridi, 2005). Moreover, she generates inner speech (Morin, 2005), she possesses an inner model of herself and external environment (Holland, 2003), she is able to anticipate perceptual and behavioral activities (Hesslow, 2002), and she acts by sensorimotor interactions with the external world (O'Regan and Noë, 2001).

Bringsjord (2007) contrasts the possibility of experiences in robots and proposes the notion of cognitive consciousness (Bringsjord et al., 2018), offering a definition in terms of formal axioms. Bringsjord et al. (2015) report the best example of cognitive consciousness by discussing a robot that passed the human test of self-consciousness proposed by Floridi (2005).

Robot consciousness is a research field aimed at two-fold goal: on the one side, scholars working in robot consciousness take inspiration from biological consciousness to build robots that present forms of experiential and functional consciousness. On the other side, scholars employ robots as tools to better understand biological consciousness.

Thus, a goal concerns the replication of aspects of biological consciousness in robots, by unifying a variety of approaches from AI and robotics, cognitive robotics, epigenetic and affective robotics, situated and embodied robotics, developmental robotics, anticipatory systems, and biomimetic robotics (Chella and Manzotti, 2009; Bringsjord and Govindarajulu, 2018).

The other goal of robot consciousness concerns the employment of robots to mark progress in the study of consciousness in humans and animals. Notably, neuroscientists involved in the study of consciousness do not exclude the possibility that robots may be conscious (Dehaene et al., 2017).

This e-book comprises a collection of 13 manuscripts published by Frontiers in Robotics and Artificial Intelligence, under the section Humanoid Robotics, on the topic on “Consciousness in Humanoid Robots.” This compendium aims at collating the most recent theoretical studies, models, and case studies of machine consciousness that take the humanoid robot as a frame of reference. However, the arguments of the articles may be applied to different kinds of robots and even to software agents.

## Overview of the Contents of the E-book

A methodological strategy for the study of robot consciousness is introduced by Reggia et al. by means of the concept of a computational correlate of consciousness. This parallels the concept of a neural correlate of consciousness in the brain. Thus, they describe a cognitive robot able to learn by imitation through low-level cognitive components such as working memory and causal reasoning mechanisms. The top-down cognitive control of the working memory of the robot is a potential computational correlate of robot consciousness.

According to Manzotti and Chella, the typical approaches toward robot consciousness as, for example, global workspace, information integration, enaction, cognitive mechanisms, embodiment, constitute the Good Old-Fashioned Artificial Consciousness. These share the same conceptual fallacy that the authors name “the intermediate level fallacy.” Thus, they outline a new conceptual framework toward robot consciousness.

The attentional mechanisms, theory of mind, and the role of emotions are all critical aspects in the study of the mechanisms underlying consciousness in humans and in robots. In this context, Graziano proposes a theory based on the attention schema as a starting point to build a conscious robot. The attention schema theory may explain how an entity lays claim to possess subjective awareness. According to Graziano, it is possible to create a robot with a rich internal model of consciousness that attributes consciousness to itself and to the people it interacts with, and that uses this attribution to predict human behavior.

Winfield proposes an artificial theory of mind that would provide robots with new capabilities related to social intelligence for human-robot interaction. The author suggests that a simulation-based internal model may offer a new basis for the artificial theory of mind. Internal models equip the robot with a model of itself and the environment, including other agents, so that the robot can test its possible actions and anticipate the consequences for itself and the other agents.

Cominelli et al. present the cognitive system SEAI (Social Emotional Artificial Intelligence) aimed for social and emotional robots designed as a bio-inspired system with a model of emotion and reasoning capabilities. In particular, SEAI comprises a simulation of Damasio's theory of consciousness.

Wang et al. and Chatila et al. consider the relevant problem of robot self-consciousness. In details, Wang et al. discuss self-consciousness in terms of NARS, an implemented general-purpose intelligent system. The authors explain how a general-intelligent system needs a notion of the “self” based on the experiences accumulated by the system during its development. The implementation of self-awareness and self-control capabilities in NARS is at an early stage; however, the overall design fits well with the processes in the human mind.

According to Chatila et al., the self-consciousness of a robot emerges by the distinction operated by the robot between its own body and the external environment. The paper proposes a cognitive architecture that considers several aspects: the perception of the robot; the interaction capabilities with the external environment; the learning phase; the interaction with other agents; the decision-making capacities.

Aspects related to architectural features for a conscious robot have been treated by Kinouchi and Mackin, Van de Velde, and Balkenius et al. In particular, Kinouchi and Mackin propose a cognitive neural architecture for a conscious robot where the primary role of consciousness is the adaptation at the system-level. The proposed architecture is based on a two-level design: the first level is related to awareness, habitual behavior, and the binding problem. The second level is associated with the general goal-directed behavior of the robot.

Van de Velde provides suggestions for robot architectures by analyzing the roles of cognitive processing and access consciousness in the brain. The author argues that consciousness is a process which is referred to *in situ* representations in the brain that underlie the possibility of cognitive access. Given this, consciousness may be related to a continuous process of cognitive access controlled by the activity of *in situ* representations themselves, as in the operations of queries and answers.

Balkenius et al. discuss the roles of memory and the inner world for a conscious robot. The authors introduce a memory model, based on neurophysiological data, that considers many aspects, such as object permanence and episodic memory. The three components of the model are an identification network, a localization network, and a working memory network. The mechanisms that fill in the sensations to the generation of perceptions can be detached from sensory input and run in isolation, allowing for planning mechanisms and daydreaming.

The active inference framework is discussed in detail by Linson et al. and by Biehl et al. The active inference framework is a bridge between computational neuroscience and robotics to psychology and phenomenology. The framework provides a theoretical basis for a unified treatment of particles, organisms, and interactive machines. The theory considers perception, reasoning, and action selection under the heading of a single principle. Notably, it suggests biologically plausible explanations for cognitive phenomena and implications for robot consciousness.

Finally, Signorelli analyses some misconceptions related to the next generations of conscious robots. The author discusses the sense in which a robot could reach capabilities at the human level, asserting that it could be possible only in case of a sentient robot. Then, a robot would be classified according to the human types of cognition. An important aspect of the author's discussion is that a conscious robot would not overcome humans but, on the contrary, it could present the very same limitations presented by humans.

## Conclusions

In summary, the advent of a conscious robot would be a tremendous scientific and technological leap.

The 13 contributions collected in this e-book touch essential aspects of the current debate about robot consciousness as the relationship between phenomenology and cognition, the role of theory of mind and self-awareness, the roles of attention and emotions, the possible problems arising from a conscious robot among us. Insights concerning the design of cognitive architectures and initial implementations are discussed. The active inference framework is investigated as a promising general theory able to consider biological and robot consciousness.

The main message from this e-book is the need for tight relationships between scientific and technological research on robot consciousness and understanding of the processes related to biological consciousness. In fact, understanding the underlying aspects of biological consciousness would greatly help to build a new generation of conscious robots, which, in turn, would contribute to a better understanding of biological consciousness.

## Author Contributions

All authors listed have made a substantial, direct and intellectual contribution to the work, and approved it for publication.

### Conflict of Interest Statement

The authors declare that the research was conducted in the absence of any commercial or financial relationships that could be construed as a potential conflict of interest.
